# Multifractal Dynamics in Executive Control When Adapting to Concurrent Motor Tasks

**DOI:** 10.3389/fphys.2021.662076

**Published:** 2021-04-16

**Authors:** Laurent M. Arsac

**Affiliations:** Université de Bordeaux, CNRS, Laboratoire IMS, UMR 5218, Talence, France

**Keywords:** multifractality, movement, motor control, variability, system complexity

## Abstract

There is some evidence that an improved understanding of executive control in the human movement system could be gained from explorations based on scale-free, fractal analysis of cyclic motor time series. Such analyses capture non-linear fractal dynamics in temporal fluctuations of motor instances that are believed to reflect how executive control enlist a coordination of multiple interactions across temporal scales between the brain, the body and the task environment, an essential architecture for adaptation. Here by recruiting elite rugby players with high motor skills and submitting them to the execution of rhythmic motor tasks involving legs and arms concurrently, the main attempt was to build on the multifractal formalism of movement control to show a marginal need of effective adaptation in concurrent tasks, and a preserved adaptability despite complexified motor execution. The present study applied a multifractal analytical approach to experimental time series and added surrogate data testing based on shuffled, ARFIMA, Davies&Harte and phase-randomized surrogates, for assessing scale-free behavior in repeated motor time series obtained while combining cycling with finger tapping and with circling. Single-tasking was analyzed comparatively. A focus-based multifractal-DFA approach provided Hurst exponents (H) of individual time series over a range of statistical moments H(*q*), *q* = [−15 15]. H(2) quantified monofractality and H(-15)-H(15) provided an index of multifractality. Despite concurrent tasking, participants showed great capacity to keep the target rhythm. Surrogate data testing showed reasonable reliability in using multifractal formalism to decipher movement control behavior. The global (i.e., monofractal) behavior in single-tasks did not change when adapting to dual-task. Multifractality dominated in cycling and did not change when cycling was challenged by upper limb movements. Likewise, tapping and circling behaviors were preserved despite concurrent cycling. It is concluded that the coordinated executive control when adapting to dual-motor tasking is not modified in people having developed great motor skills through physical training. Executive control likely emerged from multiplicative interactions across temporal scales which puts emphasis on multifractal approaches of the movement system to get critical cues on adaptation. Extending such analyses to less skilled people is appealing in the context of exploring healthy and diseased movement systems.

## Introduction

Modern conceptions of the movement system in humans are closely related to the notion of a multifractal architecture of executive control (Dixon et al., 2012; Stephen et al., 2012; Ihlen and Vereijken, 2013; Delignières et al., 2016; Bell et al., 2019). Executive control refers to the fundamental ability in humans to elaborate, maintain and adjust intentional goal-directed actions in changing environments. An effective control needs to link the brain, the body and the task environment to ensure movement stabilization through intricate interactions widely distributed among perceptual, cognitive and motor functions.

Fractal dynamics has been inferred an essential background for a typical architecture of control, providing the movement system with essential properties of flexibility (adaptability) and robustness reflected in aiming stabilization. Although counter-intuitive with the notion of movement stability, variability is inevitable in repeated motor instances. Increasingly over the past few years, researchers have demonstrated that a structured temporal variability in movement system output represents a non-trivial substrate for exploring the architecture of movement control (Diniz et al., 2011; Delignieres and Marmelat, 2012; Ihlen and Vereijken, 2013; Wijnants, 2014). Fluctuations over time are not just random errors but the output of a dynamically organized system. The identification of fractal dynamics with homogeneous sized fluctuation as a function of temporal scales (monofractal) has evolved over years to include the conception of finer multifractal properties (Ihlen and Vereijken, 2013). Multiplicative cascade models have been developed to account for multiplicative interactions within complex systems, which has inspired investigations of executive control in motor behavior (Ihlen and Vereijken, 2010, 2013; Stephen et al., 2012; Kelty-Stephen et al., 2013; Mangalam and Kelty-Stephen, 2020). Multiplicative cascading is believed to give rise to heterogeneous fractal dynamics. The degree of heterogeneity is generally associated to the degree to which multiplicative interactions shape the system architecture, which is translated in system output dynamics. Thus, the strength of interactions across scales could be captured by measures of multifractality in motor time series, as said measures index variations in fractal scaling exponents which themselves describe how fluctuations are related to observational scales.

In that respect, methodological developments have been an active part of research in fractal physiology (Chhabra and Jensen, 1989; Eke et al., 2002, 2012; Ihlen and Vereijken, 2013; Kelty-Stephen et al., 2013; Mukli et al., 2015; Delignières et al., 2016). While mostly monofractality in time series of motor behavior has depicted a global power-law linking fluctuations to observational scales, multifractal approaches have reported on the presence of several power-laws, and a more intricate system architecture, where the shape and more precisely the width of the multifractal spectrum is believed to capture the multiplicative interactions across temporal scales.

The reliability in evaluating subtle changes in complex motor behavior strongly depends on accurate determination of the shape of the multifractal spectrum. A method called focus-based approach removes certain ambiguities in spectrum determination (Mukli et al., 2015). The approach is based on computing the coordinates of a focus point which helps for a correct determination of the linear relation between fluctuations and scales on a log-log scale, whatever the range of observational scales. Interestingly the focus-based multifractal formalism holds for time-domain (detrended fluctuation analysis, DFA, signal summation conversion, SSC) and frequency-domain (wavelet leaders based on continuous wavelet transform) approaches of movement variability. As “good practices” warn on the use of multifractal methods on series smaller than 1,000 samples, a number of movement repetitions that is hardly achievable without fatigue or dropout, the fact that several methods may benefit from a focus-based approach helps strengthening applications of multifractal formalism to motor control (Torre et al., 2019). Of similar importance, the presence of multiple power-laws reflecting nonlinear rather than linear processes in time series is critical for a satisfactory understanding of the architecture in movement control (Ihlen and Vereijken, 2013). In this regard, a great deal of attention has been paid in recent years to the fact that additive linear processes, not only nonlinear multiplicative processes, may be at the origin of multifractal signature in time series. To tease apart these different sources of multifractality, it is advised to employ multifractal analyses in conjunction with a form of surrogate analysis, that provides linearized surrogate versions of original time series (Ihlen and Vereijken, 2010; Eke et al., 2012; Racz et al., 2018, 2019).

Fractal-based approaches of executive control have highlighted the role of internal sources and external sources of variation on fractal properties in movement control. Simple reaction tasks where each trial generates the same stimuli and the same kind of response is a good example where external sources of variations are minimized. By contrast, in multiple reaction tasks each trial differs to a different extent which introduces more external sources of variation. This is reflected in both the global (monofractal) scale-free behavior and the multifractal behavior of movement variability which thus allows distinguishing singular responses when either internal of external sources of variability dominate (Ihlen and Vereijken, 2013; Wijnants, 2014); behavioral time series decorrelates with increasing sources of external variations, as they did in pathological systems.

The multifractal formalism offers an opportunity to unravel effective adaptation through the multifractal architecture of motor behavior. In response to changing constraints, the motor variable will exhibit successively periods with small fluctuations to stabilize performance despite constraints and periods with large fluctuations reflecting transitions to adapt to any source of variation. The associated persistent and impersistent architecture of the motor behavior are at the origin of a wide range of local singularity exponents, which is reflected in a large multifractal spectrum (Ihlen and Vereijken, 2013). Thus, multifractal formalisms applied to movement system provides added value to explore adaptation in executive control in context of change in environmental constraints. Following this line of thought, adaptation does not resonate like an unclear general concept. It has nicely been demonstrated recently that fractal-based approach of sensorimotor variability found very interesting echo in concepts of adaptability and adaptation in movement system (Torre et al., 2019). While exploiting a multifractal approach of finger tapping time series performed with gradual sensory input deprivation, the authors showed that distinct fractal properties in motor behavior reflect impaired functional ability on the one hand, and effective internal adaptation of the movement system for maintaining performance despite constraints on the other hand.

The aim of the present study was to build on the multifractal formalism to explore movement control when adapting to dual motor tasking. By recruiting elite rugby players having developed high skills in coordinating legs and arms motor execution, a marginal need of adaptation is hypothesized, which should be reflected in preserved multifractal motor behavior.

## Materials and Methods

### Participants

Eight Bordeaux University students participated in this experiment after providing informed consent, according to the guidelines of the Faculte de STAPS and approve by the Faculte des STAPS Institutional Review Board, in accordance with the Declaration of Helsinki. Experiments were part of their academic curriculum, for which they received credits. All participants were elite female rugby players aged 21 ± 2 years, 1.64 ± 0.6 m 63 ± 7 kg. All of them have been playing rugby for more than 7 years and have reached a National or International level. They exhibited no motor impairment at the time of the experiments.

### Apparatus and Data Recordings

Participants pedaled at 60 rpm (see below) on a friction-loaded cycle ergometer (Monark 818E, Monark, Sweden) against a friction load amounting to 10 N (power output 60 W). A light meter pod connected to a PowerLab (AD Instruments) was used to detect the duration of successive pedal revolutions at a sampling frequency 1 kHz. A table was fixed in front of the participant, on which she could comfortably rest her arms while cycling.

As regards finger-tapping while cycling, a plastic box containing an iPod (Apple, Cupertino, CA, United States) was placed on the table at a comfortable distance chosen by the participant. The index finger of their dominant arm was equipped with a thimble. The iPod recorded the sound made by the index hitting the box at a sample frequency 44.1 KHz.

Circling while cycling was performed thanks to the cover of a salad spinner. Again, the light meter Pod signal served for detecting the duration of each turn. Finger-tapping and circling in single-task condition were obtained in similar conditions, seated on the cycle ergometer saddle, but the participant did not pedal.

**FIGURE 1:**
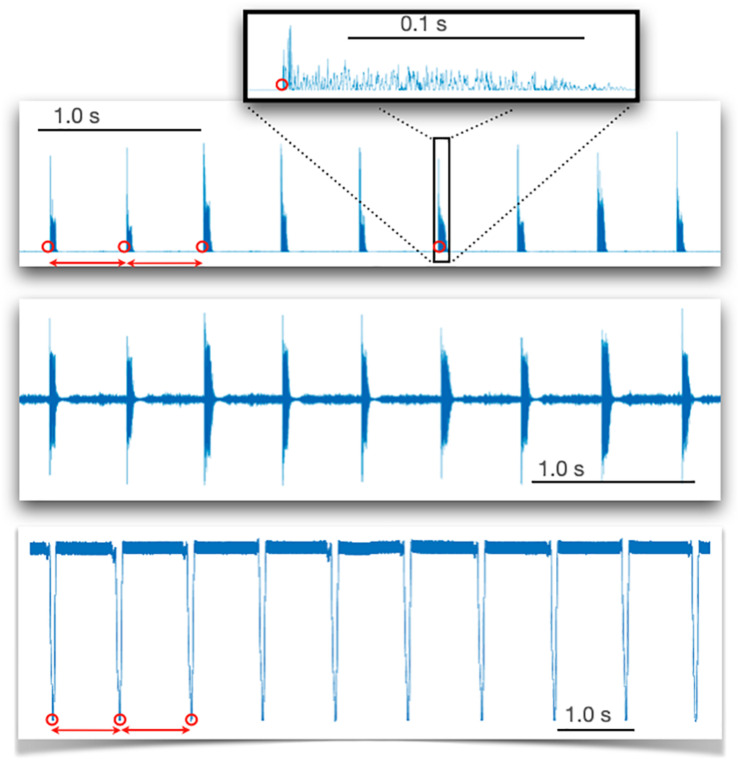
Raw signals recorded with light detection (sampling rate 1 kHz) during circling (bottom panel) and sound detection (sampling rate 44.1 kHz) when cycling and finger tapping (tapping is shown as an example). For circling, interpeak time intervals are illustrated by red dots placed on successive peaks detected by a Matlab routine. For sound wave recordings (middle panel), the sound wave was squared (upper panel), then a threshold was detected unambiguously (inset) by a Matlab routine using a threshold.

Custom Matlab (R2019b, Mathworks) routines were developed to detect peak-to-peak time intervals in each of the recorded signal (Figure 1) and to extract motor time series with high temporal resolution (given high sampling rates, 1 and 44.1 kHz).

### Experimental Design

In a randomized order [using *random*() in a spreadsheet], participants executed five runs on separate days: cycling, cycling+tapping, cycling+circling, tapping, circling.

### Experimental Procedure

Participants performed each motor task following a classic synchronization-continuation paradigm (Wing and Kristofferson, 1973). When dual-tasking, cycling was performed as the prior task. During the initial phase lasting 30 s, participants had to cycle in synch with the tempo 1 Hz imposed by the metronome. Then, the metronome was stopped and the consign was to keep the rhythm for the next 10 min. After 1 min free cycling, the metronome initialized the second motor task performed at 2 Hz, either tapping or circling. A gap between the rhythm of the concurrent tasks (1 vs. 2 Hz) was used to prevent synchronization during dual-tasking, which could be a source of particular behaviors e.g., complexity matching. When executed as a single-task, tapping and circling were stopped after 5.5 min because the aim was to obtain at last 512 repeated motor instances to get 512-sample time series, a value that was *de facto* reached after 5 min at 2 Hz.

### Experimental Time Series and Drift

**FIGURE 2:**
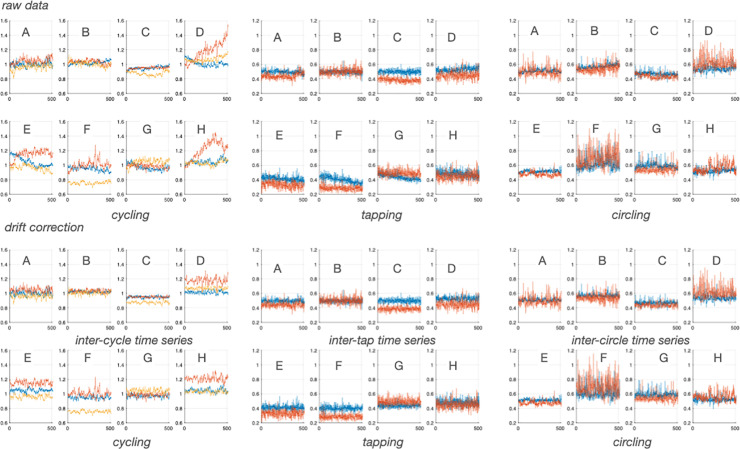
Experimental time series before (upper panels) and after (bottom panels) drift removal obtained in each participant **(A–H)**. Blue lines are single task series. Red lines are cycling+tapping in left panels and tapping+cycling and circling+tapping in right panels. Yellow lines are cycling+circling.

Drift is frequent in time series of repeated motor instances. Although any pre-processing stage applied on time series is not trivial for fractal-based analyses (Ludescher et al., 2011), it is generally advised to correct obvious drifts. Drifts in empirical signals of finite length might represent low-frequency high amplitude fluctuations that are relevant properties of the system. In power-law analyses the weight of such low-frequency fluctuations may influence the result of scaling computations, although this effect is drastically smoothed when one employed a focus-based approach to assess scaling exponents, as used here (see below). This being said, due to the finite length of empirical time series in experimental sciences, fractal characteristics are always evaluated in given ranges of observational scales (Eke et al., 2012; Ihlen, 2012). As explained below, scale-free properties were assessed in the present study using scales including 10 to N/4 samples, where N is the total number of samples (here 512). In brief, as it has been advised to remove drifts in time series for reliable analyses (Hu et al., 2001), here experimental time series were detrended by using a quadratic model fitting to the original series, which represents a very low frequency phenomenon with no fluctuating characteristics *strictosensu*, and represents signal persistence beyond the scope of control examination (Figure 2). Alternative attempts to remove the drift based on an elegant data-driven method are presented in Supplementary Material 1, but did not demonstrated successful performances.

### Multifractal Analysis

Fine temporal structures have been evidenced in most physiological signals, exhibiting scale-free (fractal) dynamics. In the multifractal formalism, it is rooted that instead of a single scaling exponent a set of scaling exponents better describe scaling in fluctuations of different sizes. The multifractal analysis is founded on characterizing scaling at several statistical moments *q*, where negative values of *q* magnify small fluctuations and positive moments *q* magnify large fluctuations (Ihlen and Vereijken, 2013). Here, characteristic scaling exponents were obtained by running a focus-based multifractal analysis on experimental series after removing drift and resizing series to 512-samples. For experimental series with *N* > 512 samples, 256 samples were selected on each side of the median sample. The method used here is based on detrended fluctuation analysis (DFA). Non-expert readers can find a detailed explanation of a fractal analysis using local detrending methods (DFA) combined with a step-by-step implementation of the routine in a spreadsheet (Arsac and Deschodt-Arsac, 2018).

**FIGURE 3:**
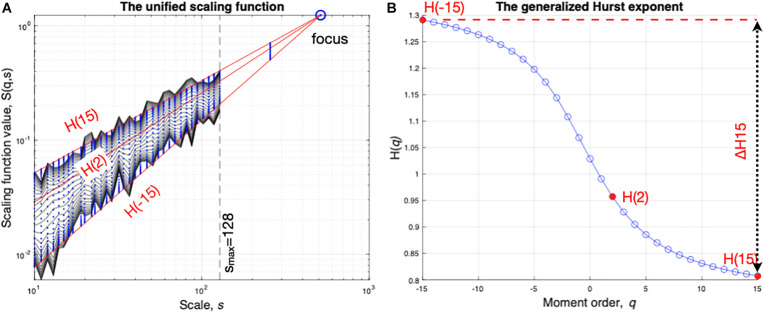
End-point parameters obtained with multifractal analysis of experimental time series (cycling as an example). The scaling function **(A)** is acquired by multifractal DFA. In that, standard deviation is calculated as a function of scale and the process is repeated along several statistical moments *q*. The generalized (*q*-dependent) Hurst exponent **(B)** is acquired *via* linear regression with the focus used as a reference point. Global scale-invariance (monofractal) is described by H(2), while the degree of multifractality is captured as the difference between H(*q*) calculated at the minimal (−15) and maximal (15) *q* moments.

In brief, DFA computes fluctuation size in linear detrended windowed parts of sample series that must have an “irregular landscape” shape, characteristic of fractional Brownian movements (fBm). Hence as a first step for most physiological signals that resemble stationary fractional Gaussian noises (fGn), the time series is cumulatively summed. Then standard deviation σ is calculated at different window sizes ranging from a minimal to a maximal scale, arbitrary chosen to cover the range of fluctuations that are under scrutinization. Here, s_*min*_ = 10 and s_*max*_ = N/4. To diminish effects of non-stationarity on fluctuations analysis, the local linear trend is subtracted in each window. The power-law dependence of σ on *s* is quantified by the Hurst exponent H according to σ(s)∝ *s*^*H*^ (Eke et al., 2012). The multifractal generalization of DFA consists in repeating the analysis by using a set of *q* moments, here *q* was an integer in the range [−15 15], to get the unified scaling function that resumes the link between fluctuations size S and scales *s* at different moments *q*:

(1) $$S\left(q,s\right)={\left\{\frac{1}{N_{s}}\sum_{w=1}^{N_{s}}\sigma {\left(w,s\right)}^{q}\right\}}^{\frac{1}{q}}$$

where N_*s*_ is the number of non-overlapping windows, each containing *s* samples, that is possible to build in the finite length time series and *w* is the index of the actual window size (number of samples) of calculation. For *q* = 0, a logarithmic averaging procedure was employed, which is a classic approach. As advised, the observational scales were evenly spaced on a log scale due to the plot that serves determining the scaling exponent by fitting to a linear model (Almurad and Delignières, 2016). As at this stage S depends on *s* and on *q*, the generalized Hurst exponent H(*q*) can be used to established their relationship according to S(*q,s*) ∝ s^*H(*^*^*q*^*^)^, which is acquired by a linear regression on the values of S(*q,s*). In other words, scaling exponents at each *q* are obtained from the slope of the fit, in the sense of minimal least-square approximation, of log S*(q)* vs. log (*s*) (Figure 3).

When applied to empirical time series the above multifractal approach sometimes yields corrupted results. The methodological developments by Mukli et al. (2015) overcome a major limitation by enforcing the behavior of the scaling function using the introduction of a well-defined focus point at the largest scale (Figure 3). They have introduced the so-called focus-based approach, using a theoretical focus of the scaling function to guide the regression for obtaining the generalized Hurst exponent *H*(*q*). In fact, on empirical time series with finite length, the regressed functions converge to one specific point termed focus, that could be included in linear computations, thus rendering the multifractal analysis of empirical time series very robust. The rationale for introducing this focus in the computational analysis can be simply shown by replacing *s* with the total length of the signal in S(*q,s*) of Eq. (1); this results in the disappearance of the sum and therefore *q* from Eq. (1). It is worth noting that similar steps leading to the characterization of the H(*q*) vs. s relationship using a focus-based approach can be followed while applying alternative time-based or frequency-based approaches of fluctuations as a function of *s* and *q*, e.g., Signal Summation Conversion (SSC) or Wavelet Leader (WL) as exposed in the seminal paper (Mukli et al., 2015). In order to strengthen the present result obtained by applying FMF-DFA, results obtained with FMF-SSC and FMF-WL following computations as in the seminal paper are provided in Supplementary Material 2.

Here, global (monofractal) scaling associated with the long-range correlations and long-term memory of the signal was captured in H(2) (Hurst exponent when the multifractal parameter *q* = 2, which is equivalent to the scaling exponent α reported in most DFA-based studies). The degree of multifractality was estimated by ΔH15 calculated as H(-15)-H(15) as a measure of how much the scaling is different for small and large fluctuations (Figure 3).

Because the FMF-DFA method systematically provides uncorrupted Hurst exponents irrespective whether the signal is a true multifractal or not, multifractality needs to be tested separately. Verification of true multifractality generally consists in the following steps (Eke et al., 2012): (*i*) identifying general scale-free behavior and the presence of long-range correlations (LCR), (*ii*) distinguishing true multifractality from background multifractal noise, and (*iii*) determining the nonlinear origin of the expressed multifractal scaling.

### Surrogate Data Testing

Multifractal scaling in empirical time series can appear as a consequence of diverse factors that falsify true multifractality. A number of falsifying sources have been identified, including heavy-tailed probability distribution, the finite size of the series, the presence of linear autocorrelations to name a few of them. Especially the length of the movement series in the present study, limited to 512 samples, calls for multiple testing to strengthen any conclusion. The procedures used here roughly follow the recommendation in Eke et al. (2012) and used in recent years (Racz et al., 2019).

As first step, to evaluate the presence of long-range correlations (LCR), the original time series were shuffled to generate surrogates (*n* = 40). Shuffling destroys all LCR thus providing white noise while preserving intact distributions of the values. The scaling exponent H(2) obtained for each original series was compared to exponents obtained in shuffled surrogates. Shuffled series have no more temporally structured variability so that the exponent of the original series must be higher if LCR truly exist in the original series. A one-sample *t*-test evaluated if the difference in the original and the shuffled scaling exponents departs from zero.

In addition, the method of Davies and Harte simulations (Davies and Harte, 1987) and ARFIMA (0,d,0) simulations were used to generate surrogate monofractal times series (*n* = 40) with equal length, variance and H(2) of the experimental time series. The multifractal index ΔH15 was quantified by using FMF-DFA in experimental and in surrogate series. If ΔH15 of the motor time series was outside the range mean ± 2σ of the ΔH15 in surrogates, the presence of multifractal noise is confirmed.

As a third step, a technique called iterated amplitude adjusted Fourier transform, IAAFT (Schreiber and Schmitz, 1996) provided phase-randomized samples of original time series while preserving essential information in amplitude, and only linear processes. IAAFT was employed here to generate surrogates (*n* = 40) of original series of cycling, tapping and circling and verify the presence of true nonlinearity multifractal phenomenon. It is expected that the width of the multifractal spectrum (ΔH15) is greater in original time series than in IAAFT-surrogates preserving only linear processes.

### Alternative (ARFIMA) Modeling

It has been advised that heuristic and/or graphical methods (like DFA) may serve as descriptive tools of fractal properties, but should rather be accompanied with alternative methods when it comes to statistical inference. Autoregressive fractionally integrated moving average (ARFIMA) modeling is a classical approach to highlight fractal properties in time series. ARFIMA are models of autoregressive moving average where the differentiation is fractional. The differencing parameter *d* is not an integer but a real number. The present analysis was limited to the most simple model ARFIMA (0,*d*,0) that is supposed to contain only long-range correlations.

The *d* parameter was estimated by using Whittle approximation of the maximum likelihood estimator thanks to the package for Matlab provided by Inzelt and served to determine the scaling component σ_*arfima*_ of the series.

ARFIMA holds only for fractional gaussian noise (fGn) and *d* is bounded within the interval [−0.5 0.5]. For fractional Brownian motion (fBm), it is possible to differentiate the series, applied ARFIMA, then estimate the fractional parameter of the fBm by adding 1 to the *d* value obtained from the fGn. The strategy exposed in Roume et al. (2019) was applied here; the best Whittle approximate of the maximum likelihood estimator by constrained optimization was found thanks to the routine developed by G. Inzelt available on the Matworks platform (G, Inzelt, 2011). When the bounded parameter *d* reached 0.49999 for a series, the series was differentiated and the new *d* obtained was converted in α value. In concrete terms, only cycling time series were concerned by the procedure mentioned here. All above computations were processed in Matlab2019b (The Mathworks, Natick, MA, United States).

### Statistics

Performance was assessed by calculating the coefficient of variation (CV, %), which is the standard error to the mean divided by the mean, and the Absolute Error (AE, ms) of time series in regards to the desired target interval (1 s at 1 Hz and 0.5 s at 2 Hz).

Each set of variables was first tested for normality with the Shapiro-Wilk test using significant level *p* = 0.05. A large majority of samples showed normal distribution, so that comparisons between samples were performed with repeated measures ANOVA with no exception to compare situations, and one sample *t*-test to analyze surrogates. It is acknowledged that ANOVA for repeated measures (within-subject factors) > 2 situations (here in cycling) are susceptible to the violation of the assumption of sphericity. Sphericity is a condition where the variances of the differences between individual differences between the situations are equal. Unfortunately, the commonly used test employed to test sphericity, the Mauchly Test of Sphericity often fails to detect departure from sphericity in small samples (here *n* = 8). As a matter of fact, no testing for sphericity was conducted prior any analysis of variance here. Statistics were performed in Matlab (The Mathworks, Natick, MA, United States).

## Results

### Performance

Performance in terms of executive control was considered to be reflected in the ability to intentionally maintain the target rhythm delivered during the initial synchronization phase. Performance was assessed by two variables, Absolute Error (AE) and Coefficient of Variation (CV, %) of the motor time series.

With regard to AE, the repeated measures ANOVA demonstrated the absence of performance degradation when dual-tasking either in cycling [AE: *F*(2, 21) = 1.62, *p* = 0.221] in tapping [*F*(1, 14) = 3.02, *p* = 0.104] or in circling [*F*(1, 14) = 1.14, *p* = 0.303].

CV provided a slightly different picture wherein CV during dual tasking increased during tapping [6.8–10.1%, *F*(1, 14) = 12.42, *p* = 0.003] and circling [6.3–10.0%, *F*(1, 14) = 5.97, *p* = 0.028]. The CV profile for cycling was similar than that of AE indicating no influence of dual tasking [*F*(2, 21) = 2.11, *p* = 0.147].

Overall, there was minor-to-no change in rhythm variability when dual-tasking was imposed.

### Testing for True Monofractality

In order to evaluate if the computed values of H(2) derived from FMF-DFA in original time series unambiguously reflect the presence of long-range correlations, the original H(2) values were compared (one-sample *t*-test) to H(2) obtained in shuffled surrogates (*n* = 40) time series. Table 1 shows main statistical results, where there is essentially no doubt for the presence of structured variability exhibiting long-range correlations, except for only 3 out of 56 experimental series, that all concern the tapping tasks.

**TABLE 1:** Results of one-sample *t*-test for the null hypothesis that the original time series has a similar α-DFA that its shuffled surrogates (*n* = 40).

H-test values	cycl	cycl+tap	cycl+circ	tap	circ	tap+cycl	tirc+cycl
Participant_A	1	1	1	1	1	1	1
Participant_B	1	1	1	0	1	0	1
Participant_C	1	1	1	1	1	1	1
Participant_D	1	1	1	1	1	1	1
Participant_E	1	1	1	1	1	1	1
Participant_F	1	1	1	1	1	1	1
Participant_G	1	1	1	0	1	1	1
Participant_H	1	1	1	1	1	1	1

*H-test = 1 means that the hypothesis is rejected, so that the original time series cannot be considered random (white) noise.*

### Monofractal Properties in Time Series

#### Scaling Exponent H(2) Derived From FMF-DFA

**FIGURE 4:**
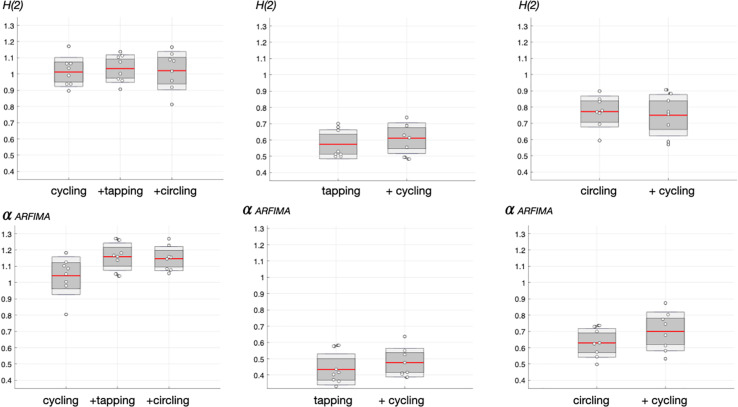
Monofractal index. Values of the scaling (Hurst) exponent in time series assessed by FMF-DFA for *q* = 2,H(2) **(top panels)** and ARFIMA **(bottom panels)**. The central mark indicates the mean, dark gray the standard error to the mean (SEM) and light gray the standard deviation. Individual values are represented by “o” symbols.

The monofractal scaling exponents H(2) obtained in each task, are shown in Figure 4. When single-tasking was compared to dual-tasking, there was no difference (repeated measures ANOVA) among cycling situations [*F*(2, 21) = 0.09, *p* = 0.912]. In the same way, there was no effect on finger-tapping fractal characteristic when performed concurrently with cycling [*F*(1, 14) = 0.67, *p* = 0.425], nor in circling when performed concurrently with cycling [*F*(1, 14) = 0.16, *p* = 0.696].

When grouped together, H(2) scaling exponents amounted to 1.02 ± 0.09 for cycling, 0.59 ± 0.09 for finger tapping and 0.76 ± 0.11 for circling. The difference across these motor-task specific exponents was highly significative [*F*(2, 52) = 97.27, *p* = 1⋅10^–18^].

#### Scaling Exponent H(2) Derived From ARFIMA

An additional precaution before interpreting above results was to fit experimental time series with ARFIMA models. Scaling exponents computed from ARFIMA modeling of the series exhibits quite similar profiles (Figure 4), which strengthens above H(2) results. Although repeated-measurement ANOVA reached overall significance [*F*(2, 21) = 3.78, *p* = 0.040], the *post-hoc* test (Bonferroni) showed the absence of difference in finer comparisons among three cycling conditions.

Clearly, tapping and circling demonstrated no change with dual-tasking, [*F*(1, 14) = 0.83, *p* = 0.379] and [*F*(1, 14) = 1.82, *p* = 0.198, respectively].

#### Testing for Multifractality

First, it was tested if ΔH(15) in original time series was outside the mean ± 2 s range of strictly monofractal surrogates generated by the Davies and Harte method on one hand, and on ARFIMA(0,d,0) on the other hand. The two methods were considered complementary given slight different properties in synthetic monofractal series reported in (e.g., Roume et al., 2019). As a criterion for excluding multifractal background noise, it was observed if one of the two methods, not each of them, provided a range of ΔH15 excluding the ΔH15 in original experimental series (Table 2).

**TABLE 2:** Comparison of ΔH15 in original time series with the average ΔH15 of strictly monofractal surrogates using either Davies and Harte simulated series (*n* = 40) or ARFIMA (0,d,0) simulated series.

*H*-test values	cycl	cycl+tap	cycl+circ	tap	circ	tap+cycl	circ+cycl
Participant_A	1/1	0/1	1/1	0/0	0/0	1/1	1/1
Participant_B	0/0	0/0	1/1	1/1	1/1	1/1	1/1
Participant_C	1/1	1/1	1/1	0/0	0/1	0/1	0/0
Participant_D	1/1	1/1	1/1	0/0	0/0	0/1	1/1
Participant_E	1/1	1/0	1/0	0/0	0/0	0/0	0/0
Participant_F	1/0	1/1	1/1	0/0	1/1	0/0	0/0
Participant_G	1/1	0/1	1/1	1/1	1/1	0/0	0/0
Participant_H	1/0	1/0	0/0	0/0	1/1	0/0	1/1

*H-test = 1 means that ΔH15 lies outside the 95% confidence interval of the surrogates, meaning that the original series is likely multifractal. H values for Davies&Harte are reported at the left of the / symbol and on the right for ARFIMA, e.g., 1/0 means multifractality is confirmed by Davies&Harte simulations but not by ARFIMA.*

Overall, Table 2 indicates that 36/56 original series (all tasks included) may be considered true multifractal behavior, distributed as follows: 21/24 (88%) in cycling, 6/16 (38%) in tapping and 9/16 (56%) in circling. Despite a low percentage of true multifractal behavior in tapping, a similar analysis was conducted further for all tasks that consisted in phase-randomization of experimental series.

Phase randomization in experimental time series (IAAFT) was performed in order to see if the observed multifractality was a consequence of true nonlinear dynamics. Table 3 indicates that 49 out of 56 (88%) of the experimental times series had nonlinear multifractal properties based on comparison (one-sample *t*-test) with their respective IAAFT phase-randomized surrogates. Nonlinear multifractality dominated in cycling (21/24 or 88%) but also reached 81% in finger-tapping (13/16) and was most obvious in circling (15/16, 94%).

**TABLE 3:** Results of one-sample *t*-test for the null hypothesis that the original time series has a similar ΔH15 that its phase-randomized surrogates (*n* = 10).

*H*-test values	cycl	Cycl+tap	Cycl+circ	tap	circ	Tap+cycl	Circ+cycl
Participant_A	1	1	1	0	1	1	1
Participant_B	1	1	1	1	1	1	1
Participant_C	1	1	1	0	1	1	1
Participant_D	1	1	1	1	1	1	1
Participant_E	1	1	1	1	1	1	1
Participant_F	0	1	1	1	1	1	1
Participant_G	1	0	1	1	1	0	1
Participant_H	0	1	1	0	1	1	1

*H-test = 1 means that the hypothesis is rejected, so that the original time series has true multifractal properties, not incidentally due to some linear structure in temporal fluctuations.*

### Multifractal Properties in Single and Dual-Tasking

**FIGURE 5:**
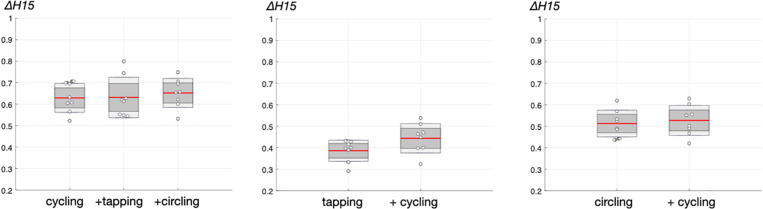
Degree of multifractality in experimental time series, as assessed by ΔH15 (see Figure 3). The central mark indicates the mean, dark gray the standard error to the mean (SEM) and light gray the standard deviation. Individual values are represented by “o” symbols.

The values ofΔH15 obtained with FMF-DFA in the experimental times series are shown in Figure 5. With regard to cycling, repeated measures ANOVA show no significant difference among single- and dual-task cycling [*F*(2, 21) = 0.21, *p* = 0.811].

Similar observations hold for finger-tapping and circling since no difference were obtained by ANOVA when comparing single- and dual-tasks [*F*(1, 14) = 3.82, *p* = 0.071] and [*F*(1, 14) = 0.20, *p* = 0.664], respectively. As a whole, FMF-DFA indicated no effect of dual motor tasking on multifractality in motor time series.

## Discussion

There are three main findings in the present study: (i) the goal of maintaining a stable rhythm during concurrent instances of cycling while finger-tapping or circling can be reached with no obvious difficulty in elite rugby players, credited with a great motor coordination; (ii) the global (i.e., monofractal) scale-free structure of temporal variability was unaltered by dual motor tasking as well, which may indicate an intact adaptive ability despite a complexified task; (iii) motor control exhibited true multifractality dominantly in cycling, whose absence of change during dual motor tasking suggests a marginal need of effective adaptation in executive control during the complexified tasks.

Overall, and despite obvious limitations (e.g., only eight participants), the present study suggests that an experimental approach based on the multifractal formalism may unravel fine adaptations in executive control and underline main properties of the movement system facing complex motor situations. The preservation of a relative stable rhythm during repeated motor instances observed here is not trivial. Experimental efforts to clarify the meaning of fractal properties may be hampered by a degraded output performance as usually observed as a consequence of aging and/or degraded functions in disease. Although it is acknowledged that age and disease are putative factors that impair complexity in the movement system (Hausdorff et al., 1997; Hausdorff, 2007; Manor et al., 2010), other factors that are more pregnant in the context of the present study have the potential to promote the emergence of a new structure in movement control. A first obvious factor lies in the external source of variation during a task, be it perceptive-cognitive or cognitive-motor. This is nicely exemplified in a review by Wijnants (Wijnants, 2014) where the gradual degree of external source of variability when comparing word naming, multiple-choice then simple-choice reaction task and precision aiming matches with a clearer fractal (1/f) scaling in time series. Precision aiming is a cyclic task (draw lines back and forth between two visual targets), with poor perturbations of input information, and demonstrated the clearest fractal organization. Apart age and disease, and excluding external sources of variations, monofractal properties have been confronted to sensory feedback deprivation during finger tapping (Torre et al., 2019). The authors concluded that monofractal properties do not reflect internal adaptations but the limit of such adaptations, it means the loss of adaptability. Building on these findings, it is suggested that the participants in the present study, maintained motor performance in each task when executed simultaneously but also keep an intact ability to adapt reflected in the unchanged monofractal architecture of executive control. One could say in addition that cycling here demonstrated the clearer 1/f scaling in agreement with previous results (Warlop et al., 2013; Gilfriche et al., 2019), which could be associated with the lowest external source of variability. This intuition is in line with the great inertia of the heavy flywheel (22 kg) of the cycle ergometer, that stores and releases kinetic energy and help thereby keeping a smooth rotation across successive movement strokes. The lowest inertia of the salad spinner (circling) and even lower inertia in the index finger (tapping) might explained gradually less clear 1/f scaling (α-DFA = 1.0) in the upper limb movement system. In finger tapping, the scaling exponent in the present study (Figure 4) was lower than the scaling exponent obtained in Torre et al. (2019), likely because of the less comfortable (elevated) rhythm (2 Hz) imposed here.

One main finding in the present study lies in unchanged scaling exponent when adapting to dual motor task (Figure 4), which may indicate an intact adaptability in executive control (Torre et al., 2019). It is important to note that even finger tapping, whose H(2) far from the ideal 1.0 exponent may indicate a more constrained movement requiring more attention and cognitive resources, did not affect fractal dynamics in cycling when participants must adapt to dual-tasking. A first reason may come from the high-motor skills in the participants, which means that future research is needed in less expert population to explore the limit of adaptability, as shown to be reached in other dual-task combining cognitive constraints with movement (Blons et al., 2019). An alternative hypothesis would posit that finger tapping do in fact hampered executive control of cycling but this is not reflected in a degraded adaptability but in effective adaptation. The present study was purposely designed to tackle this issue by exploring the multifractal signature of the experimental time series, based on previous demonstration that changes in multifractality reflects effective adaptation in sensorimotor control (Torre et al., 2019). As a main result, an absence of change in multifractality was observed in skilled participants here (Figure 5), leading to the conclusion that they demonstrated a preserved effective adaptation during each motor task, be it complexified by concurrent arms and legs movement control. The intuition that executive control is better described by multifractal non-linearity has been under the scope of many recent studies (Stephen et al., 2012; Ihlen and Vereijken, 2013; Bell et al., 2019). The issue has been addressed with the marked concern of teasing apart different sources of multifractality (Eke et al., 2012; Ihlen and Vereijken, 2013). As employed here, the combination of FMF-DFA with the analysis of surrogate data testing could bring out more clearly the existence of true multifractality and the contribution of nonlinear processes in stabilizing motor behavior. The higher ΔH15 in original time series when compared to their surrogates is an argument for inferring multiplicative interactions across temporal scales as a stabilizing factor (Bell et al., 2019). While fractal properties in repeated instances of cycling, finger tapping and circling have already been addressed, the present work takes a step further since it reveals a true multifractal architecture in the movement system and it rules out the incidental influence of linear (additive) processes (Ihlen and Vereijken, 2013; Anastas et al., 2014). Recently, an appealing study based on recording epochs in densely sampled hand movements during the Fitts Task argued that there is a so intense contribution of non-linear interactions across scales in stabilizing hand movement behavior that the wider-than-surrogate multifractal spectra index is in close relationship with aiming variability as quantified by standard deviation in times series (Bell et al., 2019).

The present results can hardly infer the exact mechanisms through which multifractality in motor behavior is preserved. The main idea is that long-range correlations arise from principles that are generic to most complex systems. Multifractality supports interaction-dominant dynamics, an intuition wherein interactions among system components are more important that components properties themselves to apprehend the system behavior. The very architecture of interactions might rely on degeneracy (Delignières and Marmelat, 2013) or on cascade dynamics (Ihlen and Vereijken, 2013), both conceptions of motor control organization being not mutually exclusive. The degeneracy hypothesis describes a degenerated network wherein components could belong to several pathways and where neighbor pathways share common components. Adopting this point of view would mean deeper degeneracy in motor control networks of elite rugby players and the presence of multiplicative cascades in control organization. They do not exhibit a simplified motor behavior after years of motor learning but true multifractality, preserved in complexified tasks. In agreement with modern theories, motor learning of performance optimization may rely on the complexification of underlying networks (Nourrit-Lucas et al., 2015).

Overall, above results leave some emerging questions unanswered. The presence of true multifractality was not unequivocal among elite athletes here (Table 2). One may want to explore individual control behavior more finely. Delignières and Torre suggested the exploitation of different modes of control along a same motor task (Delignières and Torre, 2011). They also demonstrated that the scaling exponent in finger tapping and circling is both individual- and task-specific (Torre et al., 2011). The rugby players here were selected for their high motor-skills in an attempt to get as homogenous as possible behavior to draw reliable conclusions despite a reduced number of participants. Yet, individual strategies cannot be excluded and deserve further research, that should include less skilled participants.

## Conclusion

The present study plaids for the presence of nonlinear interactions spanning several temporal scales in executive control as a reliable marker of adaptation in complexified motor tasks. The literature suggests that degeneracy (Delignières and Marmelat, 2013) and multiplicative cascade dynamics and interactivity across scales (Ihlen and Vereijken, 2013) might support control organization in movement system, which may represent a critical architecture for stabilizing repeated motor instances. Present findings extend previous knowledge on the putative role of non-linear multifractal dynamics in both control of goal-directed movement (Ihlen and Vereijken, 2013) and prospective coordination to enhance perception (Mangalam and Kelty-Stephen, 2020; Mangalam et al., 2020). As those previous observations have been made in much shorter temporal scales of densely sampled movements and depict a similar multifractal architecture, this is a strong argument for a fractal nature of movement system in the sense that similar congruent architecture properties have now been observed over a wide range of temporal scales.

Since elite rugby players with high level of motor coordination participated to the present study, the robustness in control multifractality certainly unravel a specific architecture shaped by years of physical training. Therefore, it is appealing to establish whether less skilled participants will show poorer performance in movement stabilization, a different multifractal architecture of motor control and a deeper reorganization when dual-tasking.

## Data Availability Statement

The raw data supporting the conclusions of this article will be made available by the authors, without under reasonable request.

## Ethics Statement

The studies involving human participants were reviewed and approved by the Faculte des STAPS Institutional Review Board Univ. Bordeaux. The participants provided their written informed consent to participate in this study.

## Author Contributions

LA designed the experiments, recorded and analyzed data, and wrote the manuscript.

## Conflict of Interest

The author declares that the research was conducted in the absence of any commercial or financial relationships that could be construed as a potential conflict of interest.
