# Supplementary material for: Multifractal Dynamics in Executive Control When Adapting to Concurrent Motor Tasks
Source: Front Physiol. 2021 Apr 16;12:662076. doi: 10.3389/fphys.2021.662076 (PMC8085344; doi:10.3389/fphys.2021.662076)
Supplement: Supplementary file 1 [file Table_1.DOCX]

Multifractal Dynamics in Executive Control
When Adapting to Concurrent Motor Tasks

# Drifts in experimental time series

The way in which signal stationarity is really addressed in multifractal analyses has possible consequences on the results that can be drawn. By choosing: i) how a drift in raw data is removed, a frequent phenomenon in repeated motor signals; ii) the range of selected scales within which a signal with finite length is believed to exhibit reasonable stationarity (Eke et al., 2012) or iii) the way crossover phenomenon will be apprehend in later steps of the analysis (Nagy et al., 2017), researchers take critical decisions that must be explicitly indicated. A visual inspection of raw data here leads to delete obvious drifts in some of the original time series by using a second order polynomial approximation. In any participant and in any experimental situation, it represents a fluctuation at a temporal scale that is well above s_max_. It is generally acknowledged that there exists no ubiquitous and perfect manner to manage drifts or trends in empirical signals explored through multifractal analyses (Eke et al., 2012). An appealing approach to high-pass filtering a wave in a signal that cannot be considered a mode function is to use empirical mode decomposition (EMD) or its derived improvements adding white noise to get a better separation of so-called intrinsic mode function IMF. Briefly EMD detect local maxima and minima in a signal to draw the signal envelope through spline cubic approximation of maxima/minima curves. The procedure is iterated until a wave ‘centered on zero’ can be considered an intrinsic mode function. This first IMF is subtracted to the signal and the next mode function (having thus a lower frequency) is searched by repeating the same steps. After identifying the many IMFs, the remaining part of the signal is considered a residual and may represent the drift that one wants to identify, and subtract from the experimental time series. Unfortunately, trends span more than one (residual) wave and are also localized in some (low frequency) IMFs, which do not permit to subtract trends on different signals unambiguously. The approach was attempted here and the signal ‘filtered’ by subtracting the residual are illustrated below. It is obvious that most obvious drifts, *e.g.* in subject H was not removed properly.

# Supplementary Figures


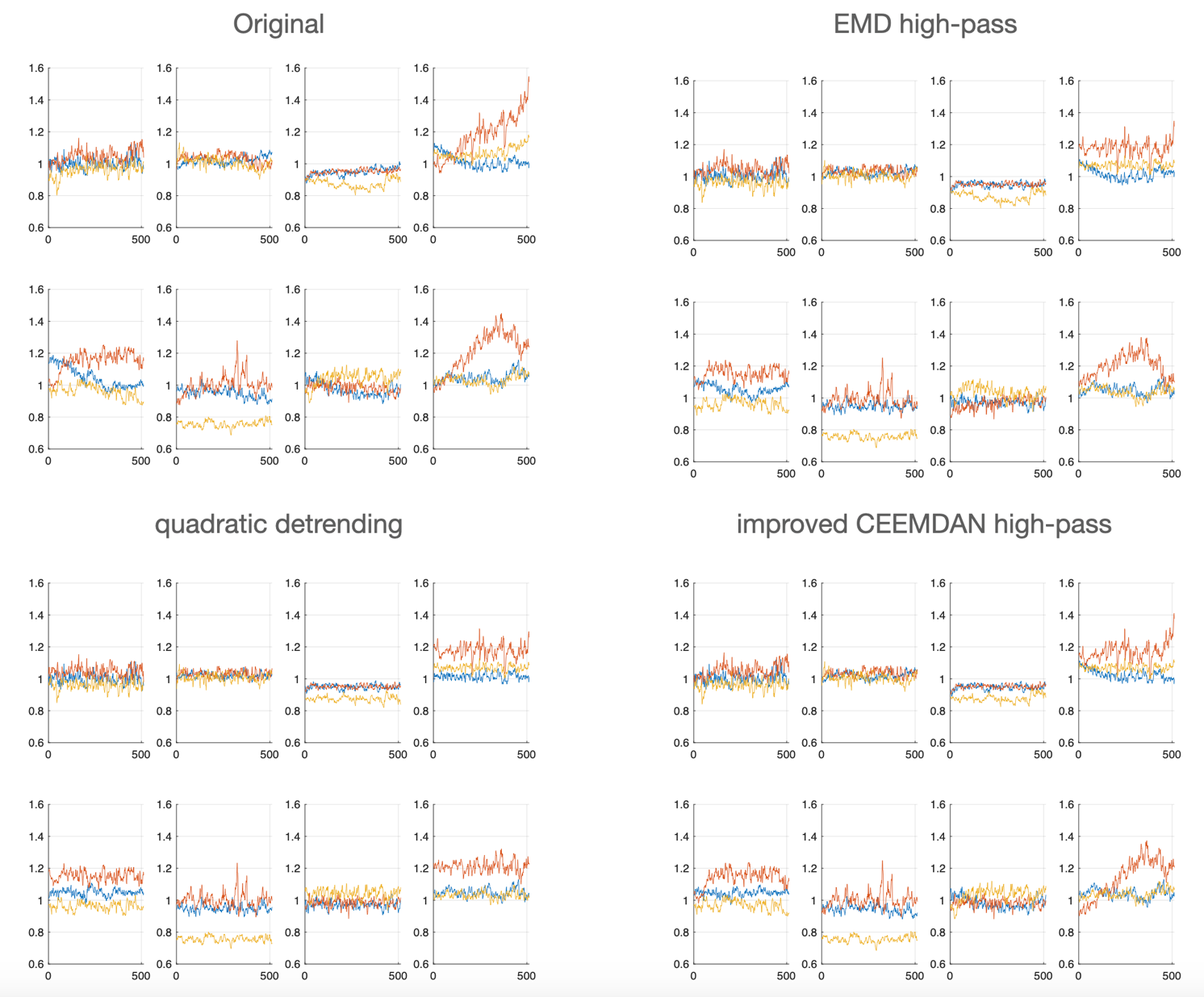


**Supplementary Figure 1.** Experimental signals before (top left) and after (other panels) different attempts to remove drifts. On panels on the right, empirical mode decomposition after removing the residual (right), either computing EMD (Huang et al., 1998) or an improved EMD (Complete Ensemble Empirical Mode Decomposition with Adaptive Noise)(Torres et al., 2011). On the left, the quadratic detrending used in the present study.

Eke, A., Herman, P., Sanganahalli, B. G., Hyder, F., Mukli, P., and Nagy, Z. (2012). Pitfalls in fractal time series analysis: fMRI BOLD as an exemplary case. *Front. Physiol.* 3. doi:10.3389/fphys.2012.00417.

Huang, N. E., Shen, Z., Long, S. R., Wu, M. C., Shih, H. H., Zheng, Q., et al. (1998). The empirical mode decomposition and the Hilbert spectrum for nonlinear and non-stationary time series analysis. *Proceedings of the Royal Society of London. Series A: Mathematical, Physical and Engineering Sciences* 454, 903–995. doi:10.1098/rspa.1998.0193.

Nagy, Z., Mukli, P., Herman, P., and Eke, A. (2017). Decomposing Multifractal Crossovers. *Front. Physiol.* 8. doi:10.3389/fphys.2017.00533.

Torres, M. E., Colominas, M. A., Schlotthauer, G., and Flandrin, P. (2011). A complete ensemble empirical mode decomposition with adaptive noise. in *2011 IEEE International Conference on Acoustics, Speech and Signal Processing (ICASSP)* (Prague, Czech Republic: IEEE), 4144–4147. doi:10.1109/ICASSP.2011.5947265.
