# Supplementary material for: Multifractal Dynamics in Executive Control When Adapting to Concurrent Motor Tasks
Source: Front Physiol. 2021 Apr 16;12:662076. doi: 10.3389/fphys.2021.662076 (PMC8085344; doi:10.3389/fphys.2021.662076)
Supplement: Supplementary file 2 [file Table_2.DOCX]

Multifractal Dynamics in Executive Control
When Adapting to Concurrent Motor Tasks

# Alternative focus-based methods to assess fractal properties

In the seminal paper by the group of Eke (Mukli et al., 2015) where a method that enforces the behavior of the scaling functions is presented, DFA, SSC and WL computations are presented, that represent as many alternatives to assess how fluctuations in time series depend on the statistical moment *q* and the observational scale *s* through the Hurts exponent H(*q,s*).

The results obtained with these alternative methods confirm those presented in the present manuscript; in brief the absence of changes in H(2) (global power-law or monofractality) and in ∆H15 (multifractality) when the tasks were complexified by concurrent motor executions.


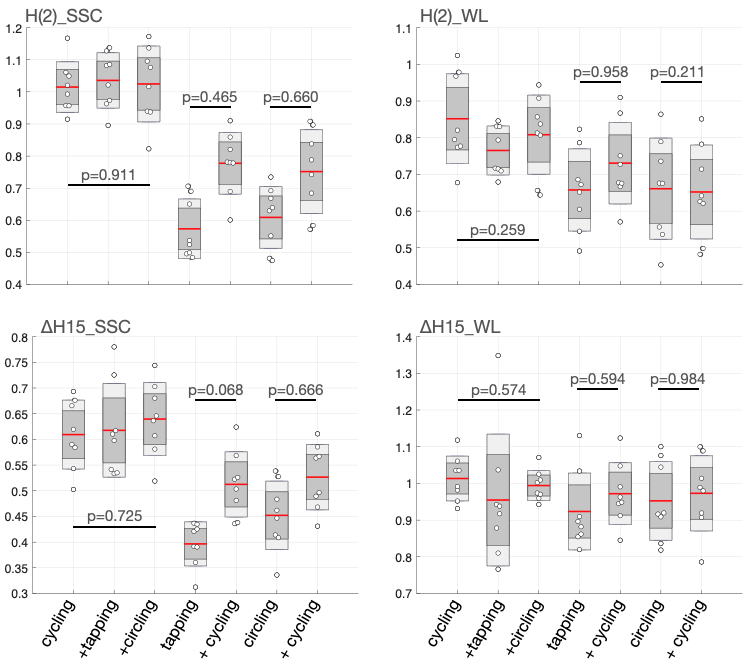


**Supplementary Figure 2.** Values of H(2) and ∆H15 metrics obtained with Signal Summation Conversion (SSC) and Wavelet Leader (WL). Statistics obtained from repeated measures ANOVA are reported.
